# Wearable bioelectronics fabricated in situ on skins

**DOI:** 10.1038/s41528-023-00265-0

**Published:** 2023-07-17

**Authors:** Faheem Ershad, Shubham Patel, Cunjiang Yu

**Affiliations:** 1 Department of Biomedical Engineering, Pennsylvania State University, University Park, PA 16801 USA; 2 Department of Engineering Science and Mechanics, Pennsylvania State University, University Park, PA 16801 USA; 3 Department of Materials Science and Engineering, Materials Research Institute, Pennsylvania State University, University Park, PA 16801 USA

**Keywords:** Materials for devices, Engineering

## Abstract

In recent years, wearable bioelectronics has rapidly expanded for diagnosing, monitoring, and treating various pathological conditions from the skin surface. Although the devices are typically prefabricated as soft patches for general usage, there is a growing need for devices that are customized in situ to provide accurate data and precise treatment. In this perspective, the state-of-the-art in situ fabricated wearable bioelectronics are summarized, focusing primarily on Drawn-on-Skin (DoS) bioelectronics and other in situ fabrication methods. The advantages and limitations of these technologies are evaluated and potential future directions are suggested for the widespread adoption of these technologies in everyday life.

## Introduction

Wearable bioelectronics has rapidly expanded into a practical solution for skin-based diagnostics, monitoring, and therapy in recent years. This class of bioelectronics enables the recording of heart rate, muscle activation, and brain activity, among many other essential physiological and physical data, while being imperceptible to and comfortable for the wearer^1–4^. Usually, the devices/sensors are designed to accommodate the general population and are prefabricated as patches that are soft, flexible, and/or stretchable for better contact with the curved surfaces of the skin. One longstanding challenge associated with such devices is motion artifacts that arise due to relative motion at the skin-electronics interface^5^. Motion artifacts are unwanted noises, distortions, or interferences in a recorded biosignal caused by the involuntary or voluntary movement of the subject or patient. These disturbances often compromise the quality and reliability of the acquired data, making it difficult to accurately diagnose, monitor, and treat various pathophysiological conditions. Another critical problem with the prefabricated devices is that they cannot be modified after their initial fabrication to accommodate the unique anatomy of each person, leading to inaccurate readings or data redundancy^6–8^. Both of these challenges are problematic across several fields, including physical/clinical medicine and diagnostics, prosthetic control, sports physiology, rehabilitation, therapy, etc. Additionally, the conventional fabrication methods based on additive manufacturing and microfabrication typically used for fabricating wearable bioelectronics require producing the devices in dedicated facilities with specialized equipment and then adhering them to target surfaces, which can be time-consuming, expensive, and may require high-temperature processes.

In situ fabrication, which involves directly fabricating devices on the target subjects with liquid/sol-gel inks, can provide simple, yet useful solutions to these issues. First, the precursor materials in their liquid/sol-gel states are much better suited to substantially increase contact area and improve adhesion with the target skins compared to their prefabricated counterparts, resulting in a stable skin-electronics interface even when the user moves. This robust interface can yield motion artifact-less physiological data, as the electronics do not move relative to the skin. Secondly, in situ fabricated bioelectronics can be customized for each individual in terms of design, precise placement, and positioning of the device at the point of care, and since the devices are made on-demand, the devices could be reconfigured for improved accuracy and reliability of measurements. Finally, the relatively simpler, rapid, and cost-effective techniques exploited in in situ fabrication make it especially well-suited for low-resource areas like socio-economically disadvantaged places, rural and remote regions, battlefields, space, and wilderness, among many others.

Recent advancements in mechanically and electrically stable materials and fabrication techniques have enabled the development of functional electronic inks that can be printed directly on skin-textured surfaces to form devices for sensing and treatment applications. Drawn-on-Skin (DoS) bioelectronics utilizes conductive, semiconducting, and dielectric inks based on composite materials^9–11^. Other conductive materials using similar in situ fabrication approaches include liquid metals^12–17^, Ag^18–22^, graphite^23,24^, or conductive hydrogels^25–28^. Since these liquid/sol-gel materials are directly applied on the skin via drawing, printing, brushing, spraying, or stamping, the liquid/sol-gel materials can flow into the crevices of skins and upon drying, they form thin films with ultra-conformal contact. This process results in the formation of a relatively stable skin-electronics interface compared to the prefabricated counterparts. In addition, these in situ fabrication methods allow the devices to remain precisely at the same location where they are originally fabricated, enabling accurate sensing and consistent treatment capabilities even when the user moves. In this review, we briefly discuss DoS bioelectronics and other materials used in in situ device fabrication, the methods of fabrication, and devices/sensors in recent reports of in situ fabrication-based technologies. The benefits of the current advances in the field, along with their limitations, are explained. Finally, potential future directions that could enable the adaptation of DoS and other in situ fabricated bioelectronics in daily life are offered.

## In situ fabricated wearable bioelectronics

In situ fabricated flexible/stretchable electronics remain rather simple compared to their prefabricated counterparts, as they rely mainly on conductive materials. Nonetheless, they are extremely useful for sensing, stimulation, and data/power transmission purposes. In this section, we describe DoS bioelectronics and some of the recent reports exploiting the aforementioned conductive materials, such as liquid/sol-gel inks for multifunctional devices and sensors. Table 1 provides a summary of the reviewed literature.

**Table 1:** In situ fabricated bioelectronics.

In Situ Fabrication Techniques/Inks	Materials	Conductivity (S/cm)	Biocompatibility	Process Strategies	Applications	Reference
DoS bioelectronics	PEDOT:PSS/Triton/Ag flakes, PEDOT:PSS/P123/Ag flakes	0.8 × 10^3^–1.5 × 10^3^	• No harmful effect to cardiomyocytes and neurons. Period: 2 weeks • No inflammation on skin tissue. Period: 3 days	Equipment/Procedure: Modified ballpoint pen Removal: Remove with towel using water	ECG, EMG, EOG, EEG, temperature, hydration, accelerated wound healing	^9–11^
Liquid metal based conductive inks	Ga-In alloy, Ni-EGaIn, EInBiTi (Field’s metal), EGaIn	1.5 × 10^3^–1.6 × 10^4^	• No cell death. Period: 72 h • No skin irritation. Period: 48 h	Equipment/Procedure: Airbrush, paintbrush, roller print Removal: Scrubbing with soap	ECG, EMG, electrical muscle stimulation and thermal treatment	^12–17,53,54^
Ag based conductive inks	Ag/PEO, AgNW, Ag/PVA	1.5 × 10^1^–1.38 × 10^4^	Biocompatible	Equipment/Procedure: Stamp based transfer process, drop casting on stencil mask Removal: Wash with warm water	Pressure sensors, NFC resonators, ECG, EMG, temperature, humidity	^18–22^
Graphite based conductive inks	Graphite	10	Biocompatible	Equipment/Procedure: Roller pen, pencil Removal: Wash with warm water	ECG, EMG, temperature, glucose	^23,24^
Hydrogel based conductive inks	Tannic acid/carboxymethyl cellulose/metal ions, Arabic gum/graphite/carbon black	2.4 × 10^−2^–4 × 10^1^	Biocompatible	Equipment/Procedure: Injection, stamping, painting Removal: Wash with warm water	ECG, EMG,EEG, blood oxygen, hydration, strain	^25–28^

A summary of the current literature on in situ fabricated bioelectronics, relevant conductive materials and their properties, process strategies, and applications.

### DoS bioelectronics

Here we review the recent developments in DoS bioelectronics. The motivation for this work is discussed, and it originates from the limitations of both conventional wearables (smart watches, fitness trackers, etc.) and wearable electronics in the form of thin patches. From this motivation, DoS bioelectronics was developed as a wearable technology platform, consisting of a library of inks that enable the development of several critical devices and sensors. The methods to evaluate the biocompatibility of the DoS inks are then discussed. Finally, the implementation of the inks to fabricate sensing arrays that can be reconfigured and customized to match the unique anatomies of human subjects is described.

#### Multifunctional, motion artifact-free sensing and point-of-care treatment

Wearable bioelectronics has quickly developed during the past 10–15 years into a practical solution for epidermal sensing. The devices are typically designed as patches, which are advantageous for making contact with the curvilinear surfaces of the skin because they are soft, flexible, and/or stretchable. However, wearable bioelectronics typically suffers from motion artifacts. Motion artifacts can cause misinterpretations and incorrect diagnoses that have serious health repercussions, which is a particular challenge for wearable health monitoring^29,30^. Misdiagnosis brought on by motion artifacts has been found in studies to considerably lower patient quality of life^31,32^. The inconsistent adhesion or imperfect conformability of the electrode to the skin during movement is the cause of artifacts that result from electrode-skin disturbances^33–35^. Particularly, mechanical disturbances at the electronics-skin interfaces are one of the main causes of motion artifacts.

**Fig. 1:**
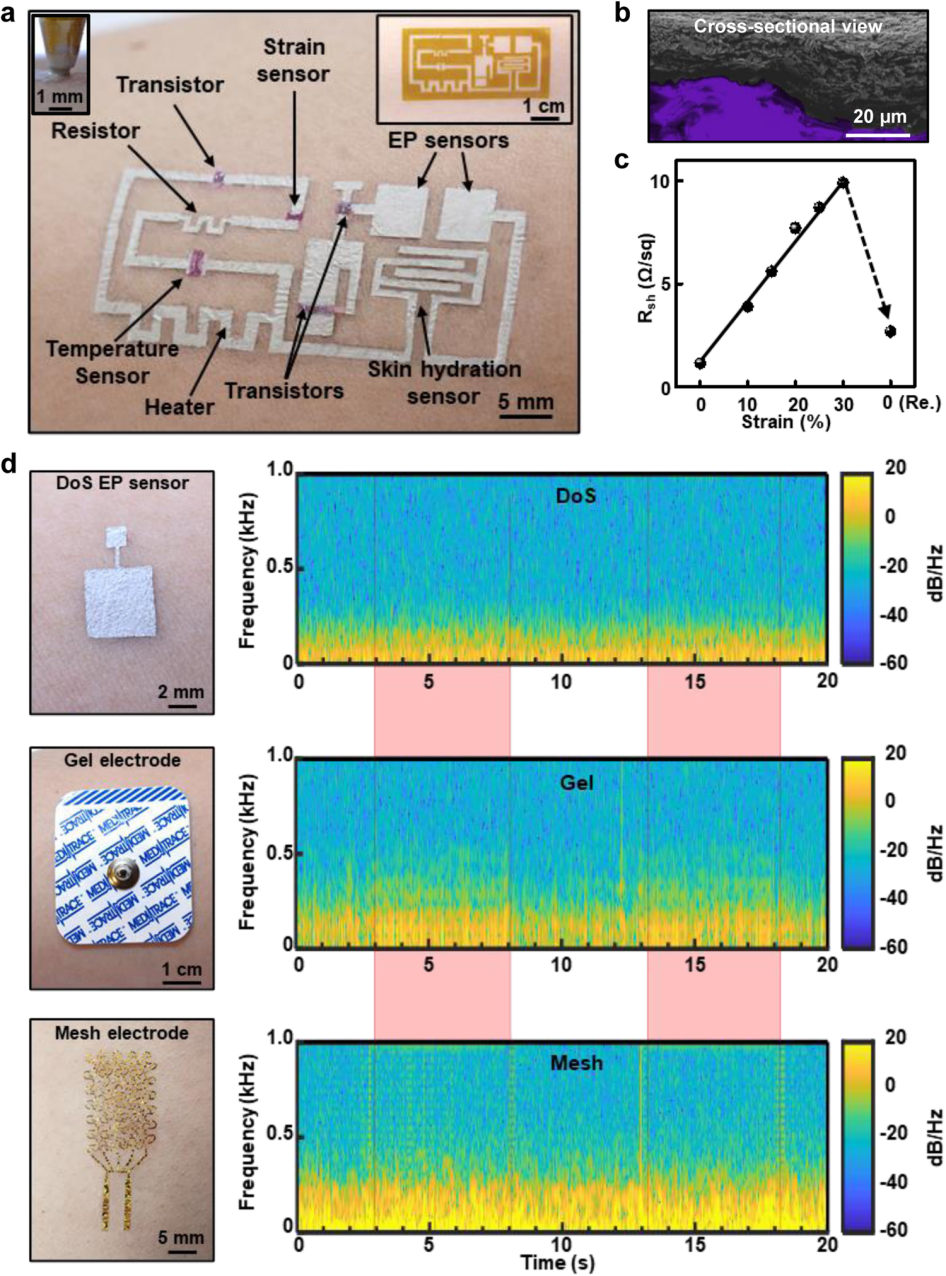
Drawn-on-skin bioelectronics. **a** An example of a DoS integrated system, which includes a resistor, transistors, heater, electrophysiological sensors, temperature sensor, strain sensor, and skin hydration sensor, is depicted on human skin. The inset shows the stencil for the conductive ink layer. **b** Cross-section showing DoS conductive ink microstructure on a skin replica. **c** Sheet resistance of the DoS conductive ink. **d** The top, middle, and bottom plots show the time-frequency maps of resting EMG signals with the addition of vibration-induced motion for DoS electrophysiological sensors, gel electrodes, and mesh electrodes, respectively. The light red bars indicate the duration of the vibrations. Reprinted with permission from Yu et al.^10^. Copyright 2020 Nature Publishing Group.

DoS bioelectronics is made by patterning (drawing) liquid functional inks using stencils onto human skin with ballpoint pens. Upon drawing, the liquid fills the crevices of the rough surface of the skin, resulting in an ultra-conformal, stretchable, and robust interface that is immune to relative motion between the skin and ink. DoS bioelectronics provides significant advantages over previous wearable and/or printed bioelectronics manufactured using specialized equipment^18,19,36–38^, including simple fabrication without dedicated equipment, capacity to deposit electronic materials on dynamic surfaces, capability to construct active electronics, multifunctionality of devices and sensors, in situ customizability and repairability, and most importantly, immunity to motion artifacts without the need for extra hardware or computation. Several DoS devices were developed including thin-film transistors, strain sensors, temperature sensors, heaters, hydration sensors, electrophysiological (EP) sensors, and electrodes for electrical stimulation as shown in Fig. 1a. The devices are based on the Ag flakes-poly(3,4-ethylenedioxythiophene)-poly(styrenesulfonate) (Ag-PEDOT:PSS) composite, poly(3-hexylthiophene-2,5-diyl) nanofibrils (P3HT-NF), and ion gel as the conductive, semiconducting, and dielectric inks, respectively. All the devices show ultra-conformal attachment to skin-textured surfaces (Fig. 1b) and the conductive ink shows a low resistance even when stretched up to 30% (Fig. 1c). Through comparisons with hospital-grade gel electrodes and the well-established (in the bioelectronics field) Au mesh electrodes, it was shown that the DoS sensors are stable during sweating, reliably capture electrophysiological signals over a long duration, show relatively strong adhesion to the skin, and are immune to motion, unlike the others.To record data from the DoS sensors, a commercially available double-sided conductive adhesive was directly placed on the DoS sensor after the conductive ink dried. A snap cable connected to the data acquisition system was laminated onto the surface of the adhesive. This wiring method was used throughout this work, including the motion artifact characterizations. Two tests, comprising local skin deformation while recording the ECG signals and arm vibration while recording the resting electromyogram (EMG) signals, were carried out to assess how the three types of sensors responded to generated motions. In each of the relevant tests, any noticeable deviations from the sinus ECG waveform during deformation or from the resting EMG potentials during vibration were regarded as motion artifacts. In the first experiment, no deviations from the ECG waveform were observed during the local stretch/compression of the skin around the DoS sensors, which was not the case for the other sensors.

In the second experiment, the effect of skin vibration (simulating shaking of the arm^34^) induced by a vibration motor (VM, applied 4 V at 1 kHz) on the baseline resting EMG potentials recorded by the DoS sensors, gel electrodes, and mesh electrodes was evaluated. It should be noted that this frequency of vibration has been shown to induce skin deflections that result in artifacts in EMG signals^34^. This is shown in the time-frequency (TF) maps in Fig. 1d. The green (indicating −10 to −20 dB) color near 1 kHz indicates that the vibration directly affected the mesh electrodes (bottom). The TF map for the mesh electrodes demonstrates that even though the VM vibrated at 1 kHz, there was a significant presence of lower frequency (>250 and 500 Hz) signals (high-intensity green color in the bottom panel of Fig. 1d during the vibration region indicated in pink) throughout the whole measured spectrum during the vibration. This lower frequency content is also present in the TF map for the gel electrodes when the VM is turned on (high-intensity green color in the middle panel of Fig. 1d during the vibration region indicated in pink) and is clearly identifiable from when the VM is turned off, demonstrating that the gel electrodes were vulnerable to vibration. For the DoS sensors, there is no change in the presence of this low-frequency content whether the motor is vibrating or not, which indicates that the vibration of the motor is not affecting the baseline resting EMG potentials. This work presented DoS bioelectronics as a simple, yet pertinent technology to solve the longstanding challenge of motion artifacts in the field of wearable bioelectronics. It also served as an introduction to the capabilities of DoS bioelectronics, demonstrating the inks, pens, and stencils as a kit that could be particularly useful in low-resource areas and eventually as a personalized healthcare tool.

#### Biocompatible DoS conductive ink

**Fig. 2:**
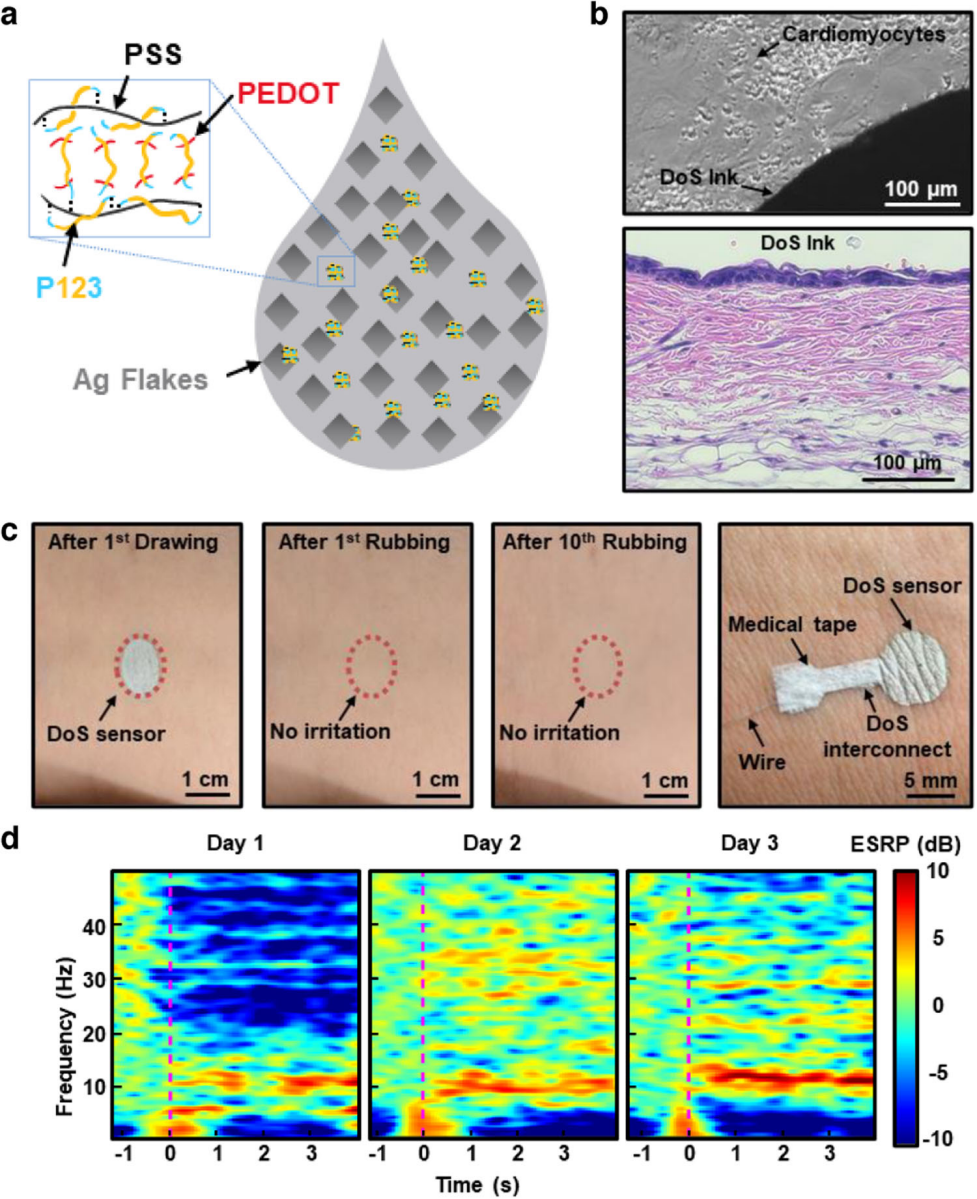
Biocompatible DoS conductive ink. **a** Schematic of one possible arrangement of PEDOT:PSS, Ag flakes, and P123 in the DoS conductive ink. **b** Microscope image of cardiomyocytes co-cultured with DoS ink (top) and skin histology of mice skin exposed to DoS conductive ink for 3 days (bottom). **c** Drawing and erasing for DoS sensors multiple times on the skin (left 3 frames). One approach to wire the DoS sensor is shown in the right frame. **d** Recording of EEG signals during mental relaxation recorded on 3 consecutive days. Reprinted with permission from Yu et al.^11^. Copyright 2022 WILEY-VCH.

As a follow-up to the first study, one aim was to clarify the biocompatibility of a modified DoS conductive ink, while studying other important electrical and mechanical properties that were not investigated previously^11^. At the time of publication, multiple studies demonstrated in situ fabrication of sensors and devices, but many of them did not thoroughly investigate the cytotoxicity and inflammatory response of their materials across cellular, tissue, and organ levels. The surfactant for the DoS conductive ink from our first study was replaced with poly(ethylene glycol)-block-poly(propylene glycol)-block-poly(ethylene glycol) (PEO20-PPO70-PEO20, Pluronic P123). This modification also proved to be beneficial in terms of the conductivity and stretchability of the DoS conductive ink. Cell viability and toxicity tests on rat neonatal cardiomyocytes and cortical neurons were carried out to show the ink’s cellular biocompatibility. The characteristic elongated shape of cardiomyocytes (CMs) could be seen in all conditions (including the control), even close to the places where the DoS conductive ink was present (Fig. 2b, top). For each condition, duplicates of each experiment were run. Cell viability studies provided additional evidence of the high cell viability (>95%) observed in the fibronectin-coated DoS ink, non-coated DoS ink, and control samples. According to the population profiles, all samples had comparable distributions. The cell density was similar to the initial seeding density (10^5^ cells/well), and the cell counts were all within the same range for both cardiomyocytes and neurons.

Next, the tissue-level biocompatibility of the conductive ink was evaluated. After a few days of wearing the ink, histological and immunochemistry tests were conducted on the skin of living mice to look for potential inflammatory markers. The conductive ink was drawn in lines on the back of CD1 mice and monitored for 3 days. Hematoxylin and eosin (H&E) staining images of the DoS ink condition over the course of three days revealed minimal to no inflammation in comparison to the control (no ink) condition. An H&E staining image after the third day of exposure to the DoS conductive ink is shown in Fig. 2b (bottom). Between the DoS conductive ink and control conditions, there were no statistically significant differences in the number of immune cells in the images from five high-power fields (HPF). The presence of inflammatory cytokines, such as interleukin-6 (IL-6) and interleukin-10 (IL-10), which are both indicators of dermatitis, was also tested for. The pathologist examined multiple HPFs and found no statistically significant differences between the control and DoS ink conditions over all 3 days, for both IL-6 and IL-10.

On human skin, no irritation could be observed after drawing and erasing the ink multiple times (Fig. 2c, left 3 frames). The creation of an alternative connection strategy (Fig. 2c, right frame) allowed for the high-quality recording of crucial EP signals such as the ECG, EMG, electroencephalogram (EEG), and electrooculogram (EOG) signals. The connection strategy relied on an insulating material (water acrylic emulsion), DoS conductive ink, medical tape, and stainless steel wire. The insulating material was drawn into a stencil, which was promptly removed. A new stencil consisting of the interconnection line and electrodes was placed and the conductive ink was drawn. Removal of this stencil, placement of a thin stainless steel wire over the interconnection line, and entrapment of the wire by the medical tape completed the connection from the DoS electrode to the data acquisition (DAQ) system. Using this setup, the EEG signals were consistently captured at a consistent time point across a number of days (Fig. 2d) falling into a range of 4–5 dB in terms of the signal-to-noise ratio (SNR), which is high for EEG signals. The EOG signals from the DoS sensors were used to operate a virtual character in a video game as a human-machine interface. These applications demonstrated that DoS sensors could be successfully used for continuous, ambulatory monitoring of wounds, skin, and other organs in future studies.

#### Reconfigurable and customizable multielectrode arrays

**Fig. 3:**
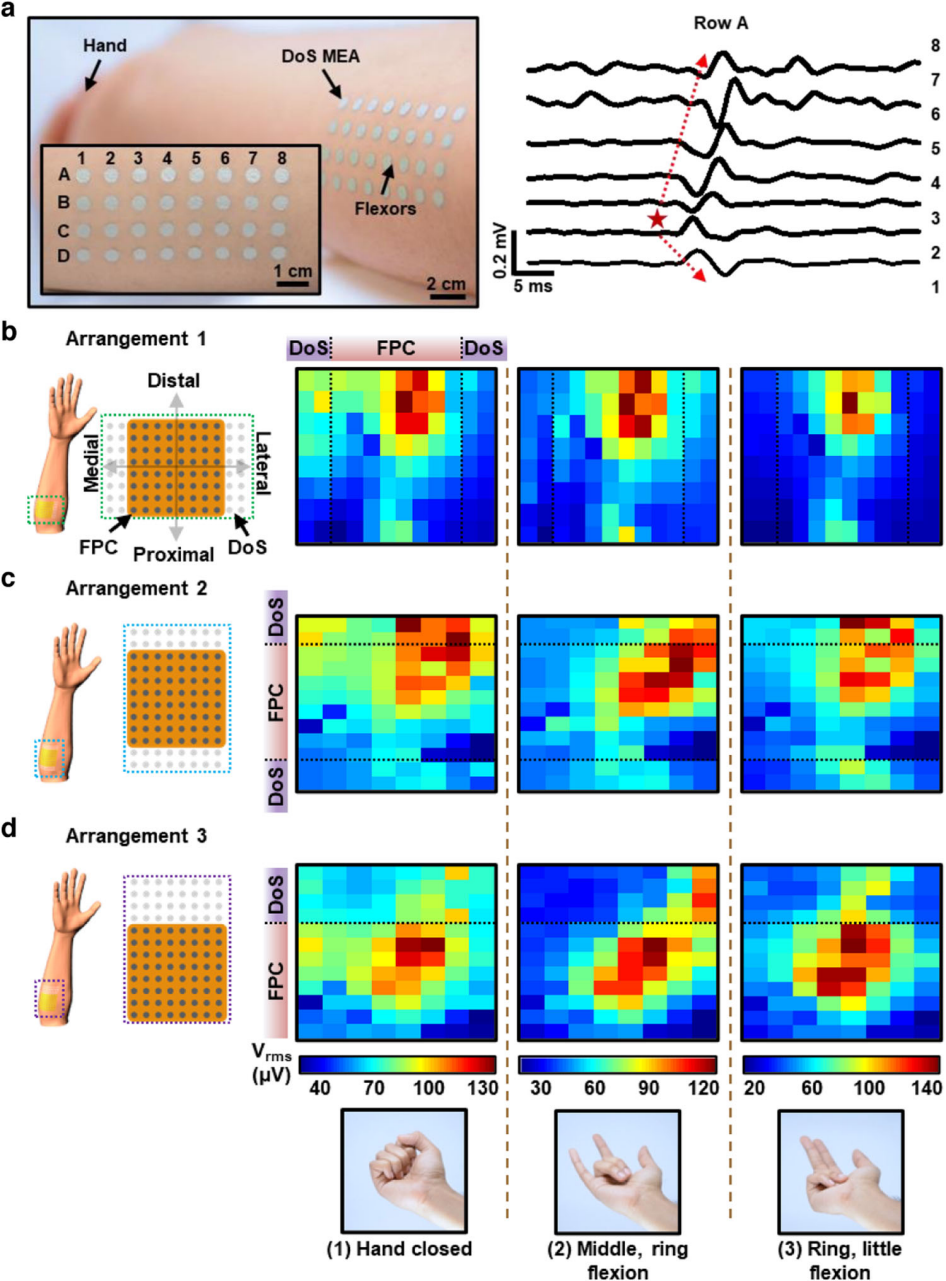
Customizable and reconfigurable DoS multielectrode arrays. **a** High density DoS MEA on the wrist flexors of a human subject (left) and motor unit propagation from one row of the MEA (right). **b** The heatmaps of the root mean square (V_rms_) of EMG signals obtained from DoS electrodes arranged in a 8 × 2 array alongside the FPC grid are shown to cover the forearm in both lateral and medial directions, aiming to concentrate the center of activity during three different hand gestures, including (1) hand closed, (2) middle and ring finger flexion, and (3) ring and little finger flexion. These gestures are displayed at the bottom of the entire figure. **c** The heatmaps of the V_rms_ of EMG signals obtained from DoS electrodes arranged in a 2 × 8 array beside the FPC grid are shown to cover the forearm in the proximal and distal directions. **d** The heatmaps of the V_rms_ of EMG signals obtained from DoS electrodes arranged in a 4 × 8 array beside the FPC grid are shown to cover the forearm in the distal direction. Reprinted with permission from Yu et al.^9^. Copyright 2023 National Academy of Sciences.

In another following study, one aim was to expand the sensing capability of DoS sensors by scaling them to cover large areas, with tuneable densities and arrangements, specifically focusing on surface EMG applications^9^. Since conventional EMG multielectrode arrays (MEAs) typically used for assessing muscle activities cannot be adjusted in situ, a trial-and-error approach is needed to identify remaining active muscles^8,39–47^. Additionally, due to the anatomical mismatch between the target muscles and the conventional EMG MEAs, relevant muscle activity is missed, high data redundancy is present, and electrode placement optimization is challenging, all leading to inaccuracies in classification algorithms. The fixed shape of those MEAs makes it nearly impossible to rearrange them to match each person’s particular muscle anatomy, which is crucial for physical medicine, prosthetic control, sports physiology, and rehabilitation research. In order to address this significant challenge, this work demonstrated DoS MEAs as a paradigm-shifting strategy. Without moving the DoS MEA, drawing new or erasing electrodes enabled on-demand tunability to fully capture the spatial extent of EMG activity and enhance classification. With advancements such as in situ reconfigurability of the devices, anatomical matching of the devices to the targets, high-fidelity mapping of EMG signals, and uniform and low-skin electrode impedance of many DoS sensors, such high-density DoS MEAs were demonstrated, marking a significant departure from our initial invention of DoS bioelectronics^10^. Most importantly, the ability of the DoS MEAs to be reconfigured to accurately match the anatomy reduced the data redundancy, enhancing the classification accuracy for prosthetic control. The DoS MEAs were fabricated via a similar approach proposed in the previous work, although many alternative options for DAQ were introduced in this work. Though several aspects of the DoS MEAs were investigated and their performance was compared to the conventional flexible printed circuit (FPC) grid, here we will focus on the high-density EMG mapping and reconfigurability of the DoS MEAs. To better understand the muscle activity and shape in both normal and pathological settings, innervation zones (IZs)—the locations where nerve terminal branches connect with muscle fibres—can be studied^48,49^. The localization of muscle IZs as prospective therapeutic targets for the treatment of movement disorders, dystonia, and spasticity is one utilization of high-density EMG^6,49,50^. The study of motor unit action potential (MUAP) propagation was used to find the IZs. The DoS MEA was applied to the wrist flexors of three subjects and was set up in a high-density configuration that matched the commercial FPC electrode’s specifications (4 × 8 electrode array, same spacing and electrode size). A potential innervation zone among the lower channels, oriented more toward the wrist, is indicated by the propagation data from row A (Fig. 3a)^51^. The average muscle fibre propagation velocity was computed from these high-density mapping results to be 6.33 m/s, and individual motor units could be detected by the DoS MEA.

In Fig. 3b, the DoS MEAs were drawn in different positions around the fixed FPC grid to determine the spatial extent of the muscle excitation during different finger flexions. Those finger flexions included (1) hand closed; (2) middle, ring flexion; and (3) ring, little flexion (as shown along the bottom of Fig. 3). In the top row of Fig. 3b, the DoS electrodes were arranged in 8 × 2 arrays to increase coverage of the circumference of the forearm. The DoS electrodes expanded the FPC grid’s spatial area and channel count. The edges of the activity recorded from the FPC grid are indicated by vertical dashed lines in the heatmaps. The EMG activity captured from the DoS electrodes is arranged in this configuration (arrangement 1) to the left and right of the dashed lines on either side of the heatmaps in the top row of Fig. 3b. The voltage maps for gestures (1), (2), and (3) display a central pattern of activity in the upper portion of the map that is more distal than proximal to the body. All the maps in the top row of Fig. 3b do not, however, depict the muscle activity boundaries and may have missed activity that is more proximal/distal relative to the mapped area. The heatmaps for arrangement 2 (middle row, Fig. 3b) reveal more information since the DoS electrodes are placed more distal than in the previous arrangement. The FPC grid edges are indicated on these maps as horizontal dashed lines (Fig. 3b, middle row). The activity detected by the DoS MEAs is shown above and below the dashed lines. Nonetheless, arrangement 2 falls short in that there is still more activity along the length of the forearm, even more distal to the existing electrode layout. In comparison to the other arrangements, arrangement 3 (Fig. 3b, bottom row) has the best configuration of DoS electrodes since it allows for a clearer localization of the centre of activity owing to improved electrode positioning. By use of these different configurations, the reconfigurability of DoS MEAs demonstrates the simplicity of revealing additional spatial information, which might be used to assess the function of muscles more accurately in both healthy and amputee patients, without substantially increasing the redundancy of the data. DoS MEAs could be used for individualized medicine in muscle therapies, myoelectric control, sports physiology, and human-machine interfaces as a large-area, tuneable-density, and in situ reconfigurable EP mapping technology.

### Liquid metal-based conductive inks

Liquid metal-based conductive inks have been the most commonly used inks among the reports of in situ fabricated bioelectronics. They have been well-reported in flexible/stretchable electronics, showing several benefits, including high conductivity, adequate printability, and biocompatibility^12–17,52–54^. However, liquid metals (Ga, GaIn, or EGaIn) alone have not been sufficient as conductive inks to be printed directly on the skin. Typically, they have been chemically/mechanically modified in order to be sintered at room temperature^14–16^. In addition, to improve their adhesion to the skin, liquid metals (LMs) have been used with other materials^13,17^.

**Fig. 4:**
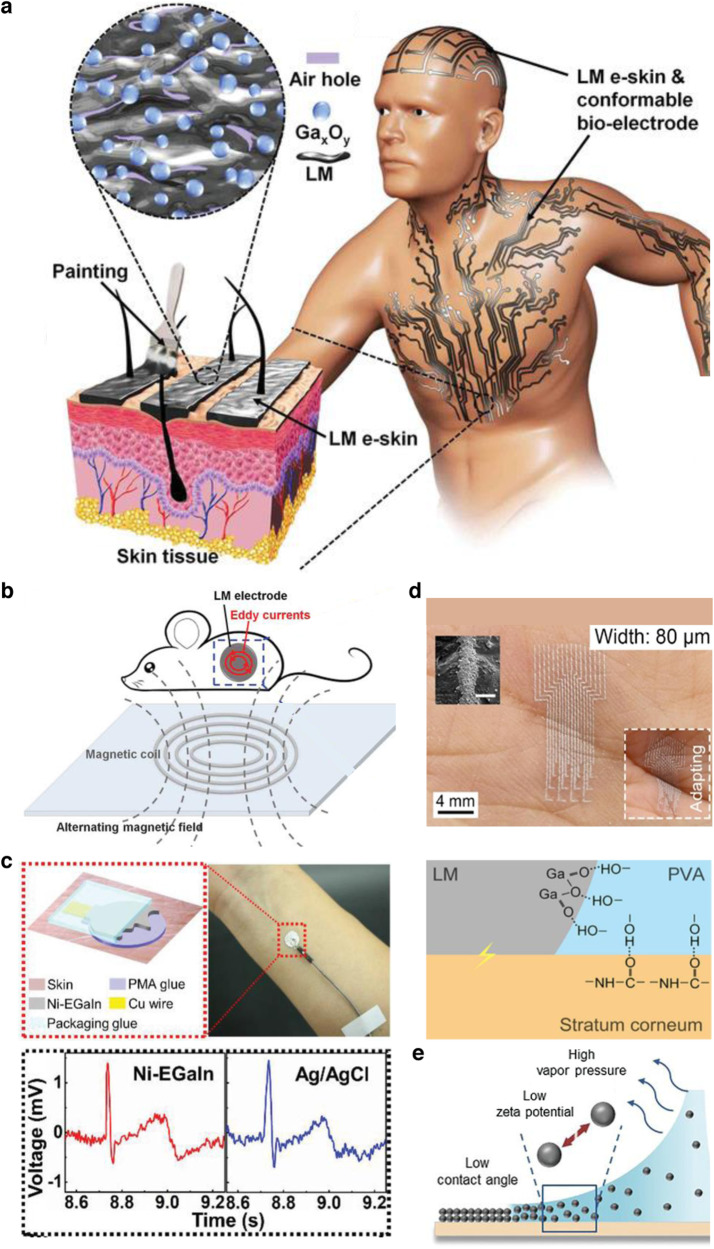
Liquid metal-based conductive inks. **a** The O-GaIn liquid metal (LM) features a uniform distribution of air holes and Ga_x_O_y_ particles, schematic of the LM in situ fabrication process, and LM as electrodes on human skin. Reprinted with permission from Liu et al.^14^. Copyright 2019 WILEY-VCH. **b** Schematic of O-GaIn conformable bioelectrodes positioned on in vivo tumors while under an alternating magnetic field. Reprinted with permission from Liu et al.^14^. Copyright 2019 WILEY-VCH. **c** Schematic of the Ni-EGaIn electrodes (top) and recorded ECG signals from the Ni-EGaIn and Ag/AgCl electrodes as a comparison (bottom). Reprinted with permission from Deng et al.^13^. Copyright 2019 WILEY-VCH. **d** Adhesive liquid metal particle (ALMP) ink micropatterned on a human hand (top) and interaction between LM, PVA, and top layer of skin (bottom). Reprinted with permission from Jiang et al.^16^. Copyright 2022 WILEY-VCH. **e** Schematic of the direct coating process using the LMP covered with Pt-decorated carbon nanotubes ink. Reprinted with permission from Park et al.^17^. Copyright 2022 WILEY-VCH.

Liu et al. proposed Ga-based LMs as conductive inks for wiring circuits and measuring the ECG signal from the surface of mice skin^12^. This study was one of the first to demonstrate a conductive ink material that could be applied directly to the skin with a paintbrush to fabricate wearable sensors. In a more recent study, Liu et al. reported an oxidized-GaIn (O-GaIn) LM ink, utilizing paint brushes to form conformal electrodes on the skin as shown in Fig. 4a^14^. The O-GaIn ink was made by stirring GaIn and exposing it to the ambient environment for over 30 min, which improved the adhesion of the ink to the skin. In addition to a simple preparation process, the O-GaIn LM ink served as an electrode for effective wireless, multisite tumor therapy in mice. By applying an alternating magnetic field through a magnetic coil, the O-GaIn electrode produced eddy currents on the skin, increased the local temperature, and ablated the tumour as schematically shown in Fig. 4b. Liu et al. also investigated the cytotoxicity of their ink in this study showing high cell viability of malignant C8161 cells after 48 hr of exposure. In this study, no masks/stencils were used to control the specific patterns of the electrodes.

In another report published around the same time by Deng et al., EGaIn was mixed with Ni microparticles to form a conductive ink that functioned as interconnections for on-skin circuits, temperature sensors, and ECG and EMG electrodes^13^ as shown in Fig. 4c. In order to pattern their ink, the authors first used a projector to project a dark image on the skin surface and then drew a layer of polymethacrylate (PMA) glue with a ballpoint pen on the skin over the dark image. The addition of the Ni microparticles improved the adhesion of the Ni-EGaIn ink to PMA glue, which served as an insulating layer between the ink and skin. The Ni-EGaIn ink was dropped and then rolled on the skin with a roller, attaching to where the PMA glue was patterned on the skin, forming the desired device/sensor. In this work, they also mention that to remove the ink from the skin, rubbing it with an alcohol wipe or washing it with soap and water was sufficient.

While the above studies did not focus so much on tuning the LM particle size, Thuo et al. explored the effect of automatic size-sorting, polydispersity of particle sizes, and transient carrier fluids on the packing of LM particles on skin or other textured substrates^15^. The researchers exploited the fact that the process of solvent evaporation results in capillary self-assembly and self-filtration, enabling smaller particles to jam into gaps formed by larger particles and improving adhesion to the target rough, textured surface. They tuned the undercooled LM core-shell particles (Field’s metal) to have a wide polydispersity, and dispersed them in methanol, water, and hydroxyethylcellulose. After homogenizing the mixture, it was applied to the target tissue via a paintbrush and chemically sintered with a chemical flux (acetic acid). Unlike the aforementioned studies using LM inks, this ink formed a mechanical bond with textured surfaces via physisorption and also enabled heat-free sintering.

Jiang et al. studied the durability of in situ fabricated devices, which was another relatively unexplored aspect of the LM inks^16^. Through the addition of polyvinyl alcohol (PVA) to their EGaIn LM, they formed an adhesive LM particle ink that had superior adhesion to skin than bulk LM. The interaction between the hydroxyl groups in PVA and the carbonyl groups of the keratinocyte layer of skin allowed for this enhanced adhesion as shown in Fig. 4d. The authors performed several important characterizations, including wear abrasion, moisture permeability, sweat resistance, and skin adhesion to emphasize the benefits of their adhesive LM ink. The ink was sprayed onto the skin, which was first laminated with an adhesive stencil to control the pattern of the devices/sensors. Their stencil design enabled the formation of conductive lines on the skin that were 80 µm wide, which is the highest resolution reported so far. They developed ECG/EMG electrodes and strain sensors to capture physiological and physical data from human subjects. Studies of the cell proliferation and migration of HUVEC cells (live/dead stain) demonstrated that the ink was biocompatible. Despite the improved adhesion, the ink could easily be cleaned by scrubbing it with soap.

Park et al. demonstrated the advantages of both self-assembly and durability shown in Thuo et al. and Dong et al., by developing an LM particle ink based on Pt-decorated carbon nanotubes on Ga LM particles, dissolved in ethanol^17^. The LM particles were made by sonicating the bulk LM. The low contact angle, low zeta potential of the particles, and high vapor pressure of the solvent resulted in rapidly formed (10 s) devices and sensors on the skin with ultra-conformability (Fig. 4e). This ink was patterned onto the skin with a brush and a laser-cut stencil on the skin surface, enabling high- resolution patterning. The authors show several relevant applications, including electrical muscle stimulators, heaters, ECG sensors, and glucose biosensors. The ECG sensors were shown to be immune to relative motion at the skin-sensor interface, which is one of the advantages of in situ fabricated wearable bioelectronics. The authors also studied the cell survival/proliferation (3T3 cells) and performed a live/dead assay to show the biocompatibility of the ink. Again, rubbing the ink with soap was enough to remove it from the skin.

### Ag-based conductive inks

Aside from LM-based conductive inks, Ag-based conductive inks also remain as another relatively common active material for direct, on-skin fabrication of wearable bioelectronics. Ag flakes, nanowires (NWs), and nanoparticles (NPs) are the typical formats found in the reported conductive inks, typically serving as conductive fillers. Like LM, Ag is biocompatible in certain formats and generally requires sintering in order to form highly conductive paths^18–22^. While not all studies consider sintering, they still show inks with sufficient conductivity to make functional devices and sensors directly on the skin. For the reported inks, the Ag-based material serves as the conductive filler either dispersed in a polymer matrix and/or water.

**Fig. 5:**
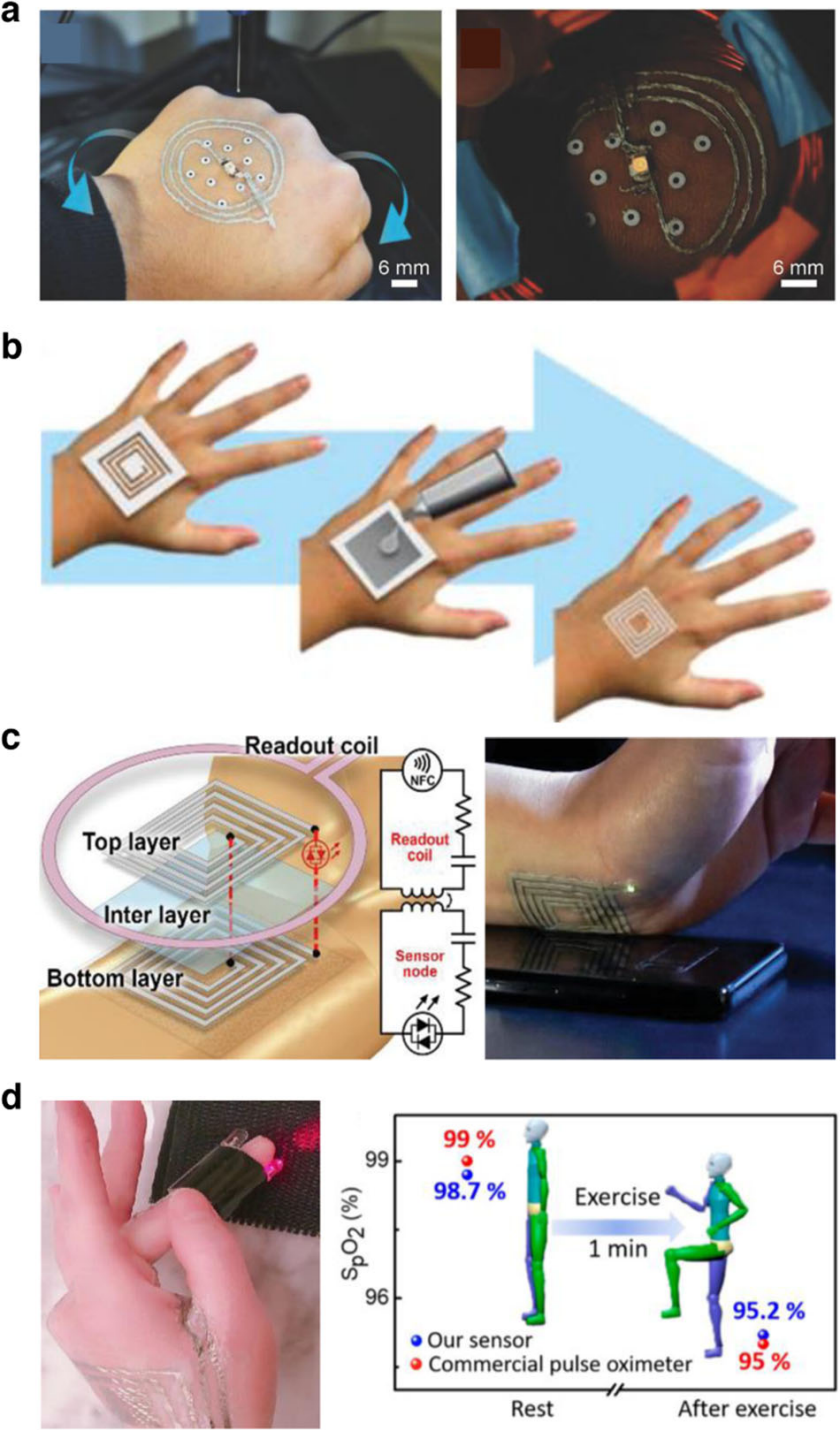
Ag-based conductive inks. **a** Image of a wireless device being adaptively 3D printed onto a human hand, allowing for unrestricted movement in the workspace (left) and image of the LED in the printed device being powered by a wireless power transmission system. Reprinted with permission from McAlpine et al.^18^. Copyright 2018 WILEY-VCH. **b** Process of patterning silver ink onto the skin using an adhesive stencil, followed by a coiled design patterned onto the index finger. Reprinted with permission from Khine et al.^20^. Copyright 2020 WILEY-VCH. **c** Schematic of the multiloop antenna (left). The electrical bridges between the terminals are indicated by red lines. The LEDs are located on the outer terminals. An example of an LED-resonator attached to the wrist is shown, powered by a device with near field communication (NFC) capabilities (right). Reprinted with permission from Khine et al.^20^. Copyright 2020 WILEY-VCH. **d** Image of a blood oxygen saturation measurement device that consists of one red LED and one infrared LED, as well as a photodiode, on the fingertip (left). Pulse rate and blood oxygen saturation levels before and after an exercise in which subjects swing their legs upward are plotted (right). Reprinted with permission from Cheng et al.^21^. Copyright 2020 WILEY-VCH.

McAlpine et al. developed an Ag flakes-based ink, mixed into poly(ethylene oxide) (PEO), water, and ethanol as the main components^18^. The PEO increased the viscosity of the ink and the ethanol shortened the drying time. The authors developed a closed-loop printing system with fiducial markers placed on the skin to direct an extrusion-based 3D printer to dispense the ink on moving freeform surfaces, such as a human hand (Fig. 5a). They printed conductive traces on the hand of a human subject and wirelessly powered an LED via inductive coupling. They also developed a wireless moisture sensor that could detect changes in impedance with different exposure durations to water vapor. The inks could be removed by simply using a tweezer and pulling the printed traces off. It should be noted that despite the easy preparation and removal processes of the ink, it is not sufficiently flexible to bend with human skin. To overcome the mechanical limitation of the ink discussed in the previous study, Franklin et al. attempted to make a more mechanically compliant variant of an Ag-based conductive ink, while maintaining a short drying time of a few minutes. Franklin et al. compared AgNPs and AgNWs dispersed in water, printed via an aerosol jet printer, and showed the AgNWs traces were more bendable than the AgNPs traces. The use of hydroxypropyl methylcellulose (HPMC) made the ink viscosity sufficient for printing. In addition, the authors compared the effect of heated (80°C) and heat-free sintering of the AgNW ink on a Kapton substrate, showing a one-order magnitude lower resistivity compared to room-temperature sintering. They showed that their ink can be used to wirelessly illuminate an LED on a human hand. Through a tape peeling test, the authors revealed that the AgNW ink had sufficient adhesion to the skin and retained its conductivity until after several tape peels, as more layers of AgNWs were removed. Removing the ink completely required vigorous rubbing with soap and water for a few minutes.

In another application, Khine et al. furthered the capabilities of Ag-based in situ fabricated bioelectronics by creating a stretchable formulation of conductive ink that could tolerate over 50% strain and showed a low resistance (tens of Ω)^20^. The conductive ink consisted of Ag flakes mixed into a nontoxic PVA glue commonly found in school supply kits. The ink was applied to the skin with a paintbrush and adhesive mask, with the authors noting that feature sizes of 1 mm or greater could be printed without any issue (Fig. 5b). They did also note that the laser cutter beam width (~200 µm) limited their mask resolution. The ink dried in 5 min on the skin surface. The authors demonstrated the first sensing capabilities with an Ag-based ink directly brushed on the skin, capturing ECG signals from a human subject with comparable quality to that of conventional Ag/AgCl electrodes. Furthermore, they showed that their Ag ink could be used to fabricate an Ag resonator for near-field communication (NFC) linkage, and the resonator could wirelessly provide power to an LED on the wrist as shown in Fig. 5c. The LED illumination varied with distance, demonstrating that power could be supplied from about 1 cm from a phone. The authors noted that once the ink was no longer needed on the skin, it could be removed by placing a layer of tape over it and then peeling it off, followed by wiping it with a wet towel.

While the previous studies show limited sensing capabilities, Cheng et al. showed a wide variety of capacitive sensors based on a Ni/Ag core/shell NPs ink and demonstrated an FPC board (FPCB) based on paper/textile to capture and transmit various EP data wirelessly with a passive sensor network^21^. They used a chemical sintering approach with the chemical agent being PVA (mixed in water) and a nanoadditive (CaCO_3_). The authors noted that the function of this sintering aid was to reduce the surface roughness of the target substrate and the required temperature for sintering. The dielectric sintering aid layer was first coated on the skin and then a stamp was dipped into the conductive ink (Ni/Ag core/shell NPs dissolved in glycol). Several resistive and capacitive devices, including temperature sensors, EP sensors, humidity sensors, and finger oximeters were constructed with conductive ink and a few conventional electronic components. The finger oximeter is shown in Fig. 5d. This is one of the first studies to present a system-level demonstration of multimodal signal acquisition with an in situ fabricated body area sensor network, consisting of each sensor with its own passive tag that could be read out with their FPCB embedded into clothing. The authors showed that the ink could be removed by washing it under running warm water, as the heat increased the solubility of the PVA in the sintering aid. In another recent study, Bao et al. demonstrated a similar ink based on Ag/Au core/shell NWs dispersed in water^22^. The ink was inkjet printed onto the hand of a human subject to form a two-terminal strain sensor for motion sensing, specifically detecting movements of the finger and hand. The resulting on-skin nanomesh was breathable and shown to be biocompatible at the cellular and tissue levels.

### Graphite-based conductive inks

While graphite is not as conductive as the aforementioned materials, it is still an excellent candidate material for in situ fabricated wearable bioelectronics as it is employed in several medical applications such as orthopaedic prosthetics, cancer treatment, and heart valves^55–61^. The reports demonstrating graphite/graphene for direct on-skin fabrication of devices and sensors are described in brief here.

**Fig. 6:**
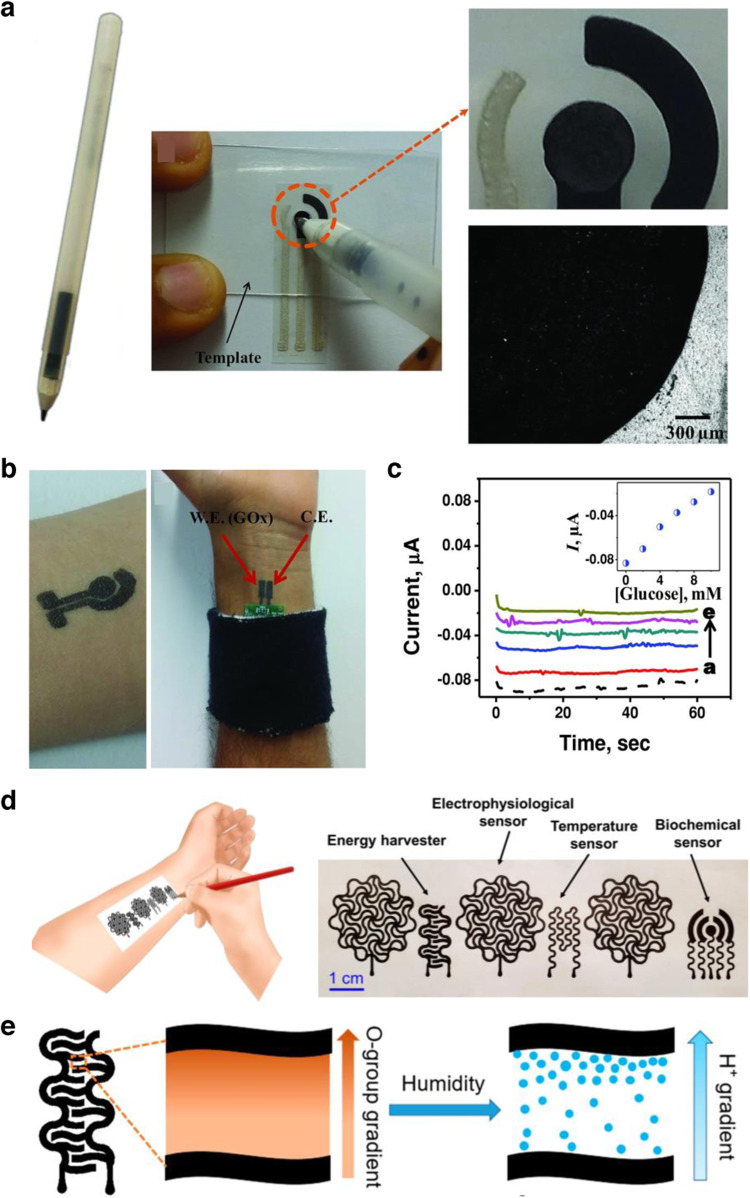
Graphite-based conductive inks. **a** Image of a roller pen filled with GOx ink (left), a drawing of the active enzyme layer created on a bare sensor strip using the GOx ink-filled pen and a template (middle), a close-up view (top right), and a microscopic image of the sensor surface with the active enzyme layer drawn by the pen (bottom right). Reprinted with permission from Wang et al.^23^. Copyright 2015 WILEY-VCH. **b** Image of epidermal glucose sensor on human skin (left), coupled with a potentiostat (right). Reprinted with permission from Wang et al.^23^. Copyright 2015 WILEY-VCH. **c** Recorded current level from administering increasing levels of glucose. Reprinted with permission from Wang et al.^23^. Copyright 2015 WILEY-VCH. **d** Schematic of the pencil-on-paper electronics drawing process (left) and various sensors (right). Reprinted with permission from Yan et al.^24^. Copyright 2020 National Academy of Sciences. **e** Schematic showing the concept of humidity energy harvesting, achieved by creating a gradient of oxygen-containing groups on cellulose paper between interdigital graphite electrodes drawn with a pencil, through the process of moisture-electric polarization. Reprinted with permission from Yan et al.^24^. Copyright 2020 National Academy of Sciences.

Wang et al. presented the first one-step fabrication of drawn-on-skin biosensors for detecting glucose, without the need for additional immobilization steps typically required for electrochemical biosensors (Fig. 6a)^23^. Their enzymatic and biocompatible ink consisted of graphite powder (conductor), polyethylene glycol (PEG, binder), chitosan (CHIT, enhances ink adhesion to the substrate), xylitol (enzyme stabilizer), and methylene green (MG, electron-transfer mediator). This biocompatible ink was further mixed with the enzyme glucose oxidase (working electrode) or used without the enzyme/mediator (counter/reference electrode) to form their electrochemical biosensor. After drawing their biocatalytic ink on the skin (Fig. 6b) with a stencil and interfacing it with a Bluetooth-enabled potentiostat, the authors successfully captured glucose concentrations ranging from 0 to 10 mM from solutions dropped on the surface of the skin (Fig. 6c). The sensor could be removed from the skin by gently washing it with soap and water.

Yan et al. further expanded the applicability of graphite-based conductive inks in their report on flexible pencil-on-paper bioelectronics^24^. Using a porous paper substrate fixed to the skin with minimal adhesive (Silbione), the authors exploited the paper as a canvas for drawing devices and sensors with a 9B graphite pencil (Fig. 6d). The authors incorporated kirigami cuts to enhance the deformability of the paper on the skin and proved that the ink on the paper remains functional after 1,000 cycles of bending. A wide variety of physiological information was obtained via capacitive ECG/EMG/EEG electrodes, and sweat biosensors (uric acid, pH, glucose). In addition, a humidity energy harvester (Fig. 6e) was demonstrated as a potential power supply mechanism for wearable sensors.

### Hydrogel-based conductive inks

Hydrogels have a wide range of implementations for bioelectronic devices/sensors and are often used as candidate sensing/stimulation materials due to their biocompatibility, mechanical softness, and deformability^4,62–65^. Though most hydrogels are not naturally very conductive, several studies utilize conductive variants or additives to enhance their conductivity. While many studies exist on conductive hydrogels, here we focus on a few of those studies specifically using conductive hydrogels for in situ fabrication of bioelectronics on skin.

**Fig. 7:**
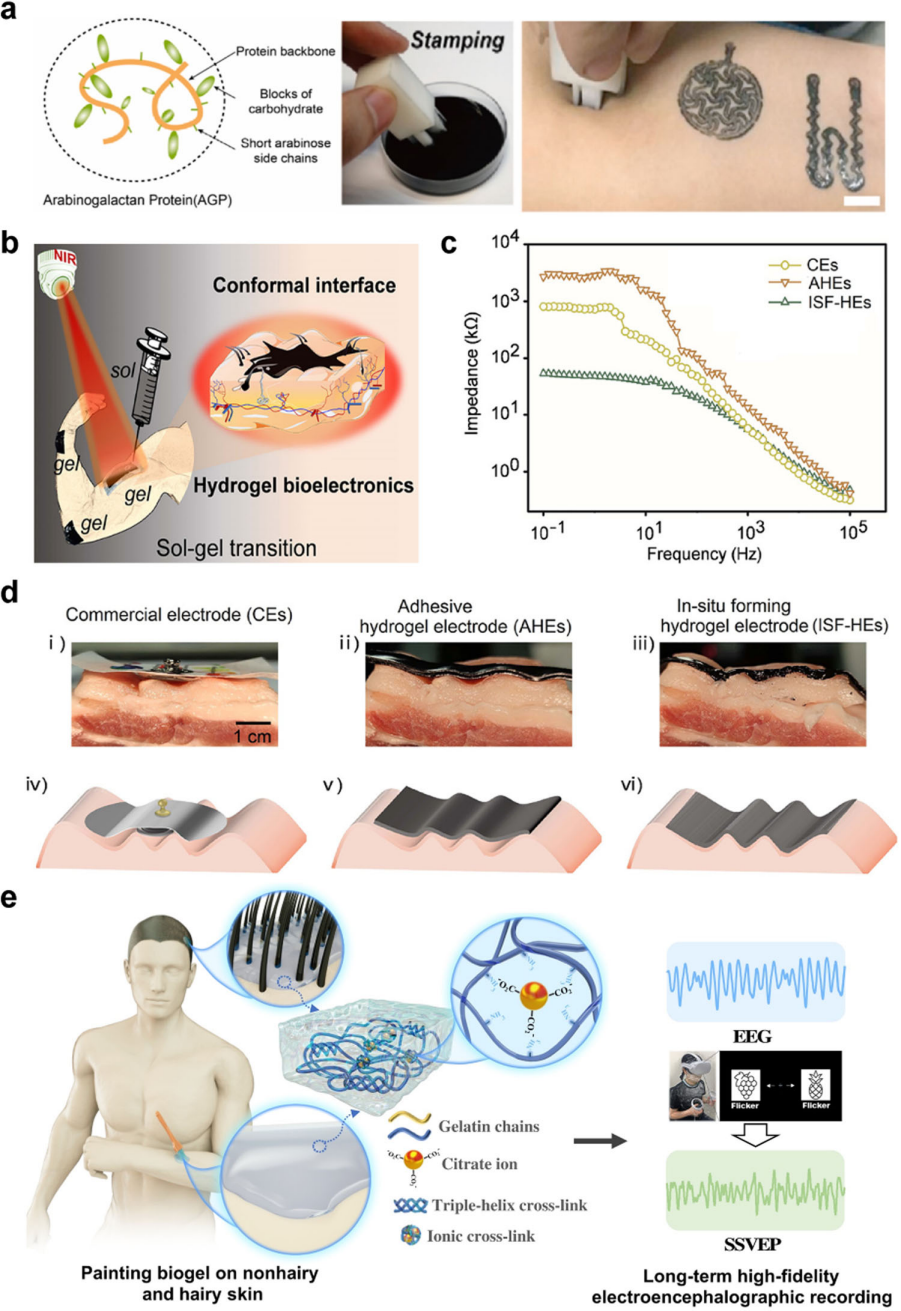
Hydrogel-based conductive inks. **a** Structure of Arabic gum protein (left), stamping (middle), and printing onto skin, scale bar = 5 mm (right). Reprinted with permission from Shen et al.^25^. Copyright 2022 Elsevier. **b** Schematic of the fabrication process using near infrared light to induce in situ cross-linking of printable hydrogels. Reprinted with permission from Zhou et al.^27^. Copyright 2022 American Chemical Society. **c** Contact impedance values measured on the human forearm, plotted against frequency (ranging from 0.1 Hz to 100 kHz), for the CEs, AHEs, and ISF-HEs. Reprinted with permission from Zhou et al.^27^. Copyright 2022 American Chemical Society. **d** Models of contact impedance measurement for the CEs, AHEs, and ISF-HEs applied to pig skin. Reprinted with permission from Zhou et al.^27^. Copyright 2022 American Chemical Society. **e** Schematic showing the paintable biogel for EEG recording on a hairy scalp. Reprinted with permission from Someya et al.^28^. Copyright 2022 American Association for the Advancement of Science.

Shen et al. developed a natural gum-based hydrogel that had water-proofing, self-healing, and easy-cleaning properties^25^. Specifically, the hydrogel conductive ink was based on the Arabic gum polymer arabinogalactan protein (Fig. 7a) mixed with conductive graphite and carbon black particles to enhance conductivity. The ink was further mixed with starch to improve ink adhesion to the skin. The ink was stamped or painted onto the skin to form electrical interconnections, ECG/EMG sensors, respiration sensors, blood oximeters, and heaters (for hyperthermia treatment). To emphasize the aforementioned ink properties, the authors showed that the ink would swell in the presence of water with a slight increase in resistance, indicating that the ink was waterproof. As much as a 1.5 mm wide damage to the hydrogel could be repaired with the addition of water on top of the damaged portion. Furthermore, the ink could be cleaned off the skin with cotton soaked in an alkaline surfactant in water. In a similar study, Shin et al. reported a cellulose hydrogel ink that was printed using a pneumatic extrusion printer onto skin to make simple circuits^26^.

Zhou et al. demonstrated that conductive hydrogel inks could even be used for motion artifact-less sensing on the skin^27^. Their conductive ink consisted of gelatin methacrylate /poly(ethylene-glycol) diacrylate, carbon nanotube, and polydopamine (GelMA/PEGDA-CNT-PDA). The GelMA served as the main carrier polymer network but when mixed with PEGDA, more cross-linking sites were available. The CNT fibres were found to be entangled with the polymer molecular chains. PDA served as an adhesion enhancer due to the high number of catechol structures. Together, all these components enabled sufficient mechanical deformability, electrical conductivity, and adhesion. The ink was applied to the skin via a syringe/3D printer into a foam tape mould already adhered to the skin. It was then crosslinked with near-infrared (NIR) light as shown in Fig. 7b. The authors demonstrate that these in situ forming hydrogel electronics (ISF-HEs) show ultra-conformal contact with the skin, unlike the adhesive hydrogels (AHEs), or commercial electrodes (CEs), plotted and pictured in Fig. 7c, d, respectively. They could detect EMG signals reliably even with skin-deformation-induced motion near the electrodes. The EMG sensors were used in conjunction with ISF-HEs-based strain sensors for an assessment of the motion and muscle activity of a subject in a shooting competition exercise. The ISF-HEs could be peeled off of the skin. Someya et al. also demonstrated a gelatine-based conductive hydrogel for EEG monitoring on the hairy scalp of a human subject over the course of 3 days (Fig. 7e)^28^. They could reliably classify steady state visually evoked potentials (SSVEPs) in a spatial attention task with their on-skin painted biogel. The biogel could be removed with water.

## Benefits and limitations

The current in situ fabricated wearable bioelectronics have been demonstrated in many critical applications, including electrophysiology, electrical stimulation, mechanical transduction, biomarker detection, wireless power, and human-machine interfaces, and do not require complex or high-temperature processes for fabrication. Some studies also investigate the effects of their functional liquid/sol-gel inks in terms of their biocompatibility. While most studies present wired modes of DAQ, a few studies show the potential use of wireless modules to capture different physiological signals or detect biomarkers. In this section, we examine the benefits and limitations of these technologies in terms of the in situ fabrication approaches, ink interactions with skin, and data acquisition.

### In situ fabrication

Here we discuss the cost/simplicity, performance/repeatability, resolution, and device functionality aspects associated with the in situ fabrication processes. Several fabrication approaches are presented in the reviewed literature, including drawing/printing, brushing, spraying, or stamping.

Compared to the costly and complex processes involved in photolithography and microfabrication for prefabricated wearable bioelectronics^2^, the aforementioned methods are fairly inexpensive and simple to use. In addition, nearly all of the methods (apart from 3D printing) do not require dedicated equipment or facilities, and even 3D printers exist in portable formats as demonstrated in McAlpine et al. and Bao et al.^18,22^. The learning curve for the aforementioned in situ fabrication methods is quite low, making them easily accessible to anyone. In addition to clinicians, surgeons, researchers, scientists, and engineers, all of whom could exploit these technologies to develop personalized devices, the general population could eventually also adopt them. Considering the cost-effectiveness, simplicity, and accessibility, the in situ fabricated bioelectronics could be especially effective in low-resource settings. However, for many of the reported technologies using pen drawing tools, clogging can be a major issue. Some strategies that can be employed to prevent clogging and ensure a continuous smooth flow can include viscosity control, particle size optimization, continuous agitation, anti-clogging nozzle design, etc.

Besides these aspects, the electrical performance and repeatable production of the in situ fabricated devices are adequate for various sensing and stimulation applications, despite the hand-based approach of most of the in situ fabrication methods^9,17^. Furthermore, since the user has full control of the fabrication process by hand, they can make devices that match the anatomy of unique subjects. From the perspective of functionality, the conductive materials discussed in this perspective are all suitable for electrophysiological, temperature, strain, and pressure sensing, as well as electrical and thermal stimulation. However, without the expensive equipment/facilities, it is challenging to develop devices with high electrical performance, uniformity, miniaturization, complexity, and functionality.

With respect to electrical performance, many of the discussed studies in this work utilize the conductive inks as-is, without any sintering, which can substantially increase their conductivity. This is primarily attributed to the fact that skin is not a suitable surface for annealing or heating. Drawing, brushing, and spraying performed by the user innately introduce variability to the device fabrication process that is not usually associated with the approaches used for prefabricated devices. This lack of uniformity or the tolerance of errors caused due to manual errors can be acceptable depending on the application. For example, in the case of electrodes used for EP sensing, they can be fabricated without the use of a stencil as the feature sizes can be relatively large and the accuracy requirements are not as strict, irrespective of variations in the drawing process on the skin such as the speed of drawing, distance from the substrate, and flow of the ink under gravity/pressure, leading to minimal variability in the skin-electrode impedance between different DoS electrodes. However, to develop transistors, the fundamental building blocks of electronic circuits, it is critical to develop features with high precision, resolution, and uniformity. Variations in the thickness direction can make it exceptionally difficult to make transistors simply by drawing. Currently, many of the devices are limited in their feature size due to the restrictions of the drawing tool (pen, syringe nozzle, brush bristle width, etc.) and the mask resolution. Fabricating electrodes with millimetre dimensions is simple, but for a transistor, forming a channel length that is tens of microns across (directly on the skin) is not trivial. This is mainly due to the stencil adhesion to the skin and the tiny features of the stencil being too small to be cut by a cutting machine (blade, laser, etc.). Finally, since nearly all the reports of in situ fabricated bioelectronics use only conductive and/or dielectric materials, the functions of the devices are primarily limited to passive operation. Aside from our first report on DoS bioelectronics, there are currently no skin-compatible semiconductor inks, which would enable the creation of transistors, logic gates, and eventually integrated circuits.

### Ink interactions with skin

Ideally, the desired result from the in situ fabrication processes is a thin film that is fully cured on the skin surface and forms ultra-conformal contact. Some important properties of the printed inks when applied to the skin include biocompatibility, adhesion, breathability, sweat resistance, and washability. Furthermore, the ink should be able to be used on any skin surface, hairy or glabrous skin.

Many of the studies discussed in this perspective report the biocompatibility of their inks. Studies of cell viability, cell growth and proliferation assays, lactate dehydrogenase production, immune cell count, and skin histology have revealed that the conductive inks do not cause substantial reductions in cell health, nor do they induce inflammation. Compared with prefabricated wearable bioelectronics, in situ alternatives can offer greater adhesion to the skin without the use of additional adhesives^10,19,20^. This is due to the liquid/sol-gel nature of the inks, which can flow into the cracks of skin, increasing the contact area and thus improving adhesion. In addition, the inks could potentially form hydrogen bonds with the surface of the skin, whereas the majority of the prefabricated bioelectronics solely have van der Waals interactions with skin^16^. Multiple reports also look at the air/gas or water permeability, demonstrating that the resulting films are breathable^17,22,27^. One study also developed a sweat model to mimic the actual sweating scenario on the skin, which could serve as a basis for studying sweat effects^16^. A few studies also evaluate the sensor performance during sweating^10,16,21,25,28^. The inks also have been shown to be easily washable, mainly removed by soap, water, and paper towels. Finally, the inks can be dropped onto the surface of both hairy and glabrous skin while remaining conductive despite the discontinuities from the hair follicles^28^. For prefabricated bioelectronics, it is nearly impossible to laminate them on hairy areas as the hair gets in between the device and skin.

Despite the reported studies showing the biocompatibility of the resulting devices, there is no study of the long-term effects of ink exposure on the skin. The longest study reports exposure after 3 days^11^. In addition, none of the studies discussed in this perspective check for any uncured materials or penetration of particles into the skin. Most evaluations are macroscopic, revealing that nothing can be clearly observed on the skin to the naked eye. In this case, the prefabricated devices may be more suitable to ensure that no harmful compounds leech out of the device. Considering the adhesion property, not all the reported studies evaluate the interaction between the constituent materials and skin. Some of the in situ fabricated films also do not form a continuous film, so removing them is not as simple as just peeling off the device. Not all the discussed papers examine the breathability of their in situ fabricated devices, but it should be studied in order to ensure that the process of sweating is not affected. More careful analysis of the effects of sweat on device performance is also necessary, especially if the inks are based in water. In terms of removing the ink, most studies have not examined if any materials remain on the skin after washing thoroughly. Lastly, it is still challenging to pattern devices on hairy skin even with the current in situ fabrication approaches, though some of them could be adapted to do so.

### Data acquisition

One of the critical parameters for data acquisition from in situ fabricated sensors is skin-electrode impedance. Considering the unique advantage of in situ fabrication methods, which enable ultra-conformal and high-surface-area contact, skin-electrode impedance could be reduced by 1–2 orders of magnitude compared to the conventional wearables and prefabricated wearable bioelectronics^27^. Another factor that is not extensively explored is the wiring aspect of in situ fabricated sensors. A majority of the prefabricated wearable bioelectronics can exploit the conventional wired DAQ methods, using breadboard/alligator wires, or anisotropic conductive film cables and PCBs, as examples. In addition, they can take advantage of FPCBs or wireless modules that can even be made on the same substrate as that of the device. For in situ fabricated wearable bioelectronics, wiring and wireless approaches for DAQ is not always trivial.

Most of the reports of in situ fabricated wearable bioelectronics show wired connections that are fixed via adhesives or glues to the skin for sensing purposes^9–14,16,17,25^. Approaches for this hard-soft connection include adhesives like conductive ARclad® 8001–77, TD23, etc. with button connectors, thin wires, etc. for electrical connection. These are sufficient for demonstrating that the sensors/devices are operational. A few of the studies also show wireless approaches that involve placing FPCBs or wireless modules in contact with the in situ fabricated interconnection lines^21–23^.

The current in situ fabrication approaches still face a challenge in wiring as the hard-soft contact between DAQ wires and skin is unfavourable. This connection is a potential failure point for proper device operation. A potential solution for obtaining reliable hard-soft connection is by use of sticky liquid metal conductors (SLMC), composed of liquid metal enriching the surface and a pressure-sensitive adhesive matrix to adhere to different surfaces, forming both electrical and mechanical connections without involving high-temperature and organic solvents that may damage human tissues^62^. Alternatively, the wireless approaches could be better, but they still require connections to conventional wireless data transmission components which are rigid, again leading to the hard-soft interface. If wireless operation while minimizing the hard-soft interfaces is successfully achieved, the daily usability of these technologies becomes substantially more plausible.

## Outlook

We discussed the recent reports of in situ fabricated wearable bioelectronics and their benefits and limitations. In this last section, we provide potential future directions for this field from the perspective of in situ fabrication, ink interactions with skin, and data acquisition.

In situ fabricated bioelectronics are largely limited to conductive materials. Still, improving the current conductive materials and exploring other alternatives such as LM droplets could be crucial for in situ drawing in applications such as physiological sensing and actuation^66,67^. More importantly, developing printable and biocompatible insulating, interfacing, semiconducting and dielectric materials/inks^10^ to enable additional functionalities should be a priority. For example, hydrogels have been used and successfully demonstrated to reduce or entirely remove the effect of mechanical noise during sensing, also resulting in motion artifact-less data^68^. Efforts to do so will increase the library of inks available to all to use. Another important point from this aspect is the education of the target users to be able to develop devices/sensors on their own for either themselves or their patients/subjects. Clarifying the detailed steps of the in situ fabrication will enable individuals with any background to customize the devices for each application. Although some studies examine the stability or usability of their conductive ink over time, it is still necessary to clarify the shelf-life.

Many of the above-reported in situ fabrication technologies rely on human user operation, which can be error-prone. There is a need for standardizing the characterization of in situ fabrication approaches to reduce some of these human errors. Rigorous bench testing as performed for conventional wearables and wearable bioelectronics could be performed by exploring the various combinations of parameters (e.g., speed of writing, distance from the substrate, performance under deformation, etc.) in the lab setting after fabricating the devices in situ. Clear protocols can be established for each in situ fabrication technique that includes a step-by-step guide for the procedure, including the materials, equipment, and settings used. This will ensure that the same process is followed each time, reducing variability between experiments. Factors such as ink viscosity, ink spreading, surface treatment, room-temperature sintering, and drying time (as a few examples) should be thoroughly investigated. Also, in situ fabrication approaches should be validated through experiments on a diverse range of skin types to ensure that they work across different skin types and conditions and test their reproducibility, sensitivity, and specificity. Another aspect that needs to be considered for standardization is the contact impedance between the device and skin as it can influence the output signal significantly, and can vary from skin to skin, area of contact of the device to skin, etc. The safety of in situ fabrication techniques also needs to be evaluated to ensure that they do not cause damage or harm to the skin. This can include conducting standard cytotoxicity assays, histological analysis, and other safety assessments.

Another remaining direction for studying the ink interaction with skin involves revealing the effect of the natural production of oils. For example, in more hairy areas of skin (e.g., scalp), sebum production will be higher and potentially cause changes in the operation of the in situ fabricated devices. Furthermore, as new skin arises to the top of the epidermis and dead keratinocytes fall off, the effect on the in situ fabricated devices remains unknown. A final important point involves the study of cell death and inflammation in the case of solvents/materials that are known to be skin irritants. Despite the safety data sheet recommendations, it is also important to consider the concentration, volume, time of exposure, and method of exposure when deciding the material constituents of the electronic inks.

For data acquisition, there have yet to be any long-term studies under ambulatory conditions with in situ fabricated wearable bioelectronics. These types of studies could also clarify how natural oil production and skin renewal occurs in the presence of an in situ fabricated device, as mentioned above. Another possible direction is to develop fully in situ fabricated DAQ systems, without any conventional PCB components. Although it may be challenging to develop all the circuitry on the skin, there is some promise in this direction as some studies have shown passive tags as one approach for wireless data collection^69^.

In this perspective, based on reviewing the capabilities of in situ fabricated devices and sensors, their benefits and limitations, and on discussing potential future directions, we strongly believe that the development of drawing tools, inks, and components will enable the formation of kits that can be used by anyone, allowing for truly personalized healthcare devices.

## Data Availability

The authors declare that all data supporting the findings of these studies are available within the paper.
